# A bibliometric analysis on discovering anti-quorum sensing agents against clinically relevant pathogens: current status, development, and future directions

**DOI:** 10.3389/fmicb.2023.1297843

**Published:** 2023-11-30

**Authors:** Bo Peng, Yanqun Li, Jiajia Yin, Wenping Ding, Wang Fazuo, Zhihui Xiao, Hao Yin

**Affiliations:** ^1^Institute for Environmental and Climate Research, Jinan University, Guangzhou, China; ^2^CAS Key Laboratory of Tropical Marine Bio-resources and Ecology, South China Sea Institute of Oceanology, Chinese Academy of Sciences, Guangzhou, China; ^3^Southern Marine Science and Engineering Guangdong Laboratory (Guangzhou), Guangzhou, China; ^4^Sanya Institute of Ocean Eco-Environmental Engineering, Sanya, China

**Keywords:** antimicrobial resistance, anti-quorum sensing, biofilm, drug discovery, inhibitor, quorum sensing inhibition, virulence

## Abstract

**Background:**

Quorum sensing is bacteria’s ability to communicate and regulate their behavior based on population density. Anti-quorum sensing agents (anti-QSA) is promising strategy to treat resistant infections, as well as reduce selective pressure that leads to antibiotic resistance of clinically relevant pathogens. This study analyzes the output, hotspots, and trends of research in the field of anti-QSA against clinically relevant pathogens.

**Methods:**

The literature on anti-QSA from the Web of Science Core Collection database was retrieved and analyzed. Tools such as CiteSpace and Alluvial Generator were used to visualize and interpret the data.

**Results:**

From 1998 to 2023, the number of publications related to anti-QAS research increased rapidly, with a total of 1,743 articles and reviews published in 558 journals. The United States was the largest contributor and the most influential country, with an H-index of 88, higher than other countries. Williams was the most productive author, and Hoiby N was the most cited author. Frontiers in Microbiology was the most prolific and the most cited journal. Burst detection indicated that the main frontier disciplines shifted from MICROBIOLOGY, CLINICAL, MOLECULAR BIOLOGY, and other biomedicine-related fields to FOOD, MATERIALS, NATURAL PRODUCTS, and MULTIDISCIPLINARY. In the whole research history, the strongest burst keyword was cystic-fibrosis patients, and the strongest burst reference was Lee and Zhang (2015). In the latest period (burst until 2023), the strongest burst keyword was silver nanoparticle, and the strongest burst reference was Whiteley et al. (2017). The co-citation network revealed that the most important interest and research direction was anti-biofilm/anti-virulence drug development, and timeline analysis suggested that this direction is also the most active. The key concepts alluvial flow visualization revealed seven terms with the longest time span and lasting until now, namely *Escherichia coli*, virulence, *Pseudomonas aeruginosa*, virulence factor, bacterial biofilm, gene expression, quorum sensing. Comprehensive analysis shows that nanomaterials, marine natural products, and artificial intelligence (AI) may become hotspots in the future.

**Conclusion:**

This bibliometric study reveals the current status and trends of anti-QSA research and may assist researchers in identifying hot topics and exploring new research directions.

## 1 Introduction

Antimicrobial resistance has become a global public health threat, and finding new anti-infection strategies to control the spread and harm of resistant bacteria is an urgent task. In recent years, attempts to develop new classes of antimicrobial agents have included targeting specific virulence factors or virulence regulation mechanisms, rather than cell survival, to reduce the selective pressure that leads to the emergence of resistance of clinically relevant pathogens. One such strategy is to interfere with quorum sensing (QS) (Soukarieh et al., 2018).

QS is a communication mechanism among bacteria, involving the synthesis, release, diffusion and perception of small signal molecules, to coordinate gene expression regulation, cope with environmental changes, and optimize survival strategies. When the number of bacteria reaches a certain threshold, the concentration of signal molecules in the surrounding environment reflects the bacterial density, thereby synchronizing the transcription of multiple genes and achieving collective behavior of bacterial populations. QS plays an important role in bacterial physiology and pathogenesis, such as controlling biofilm formation, virulence factor production, antibiotic resistance, metabolic adaptation, etc. (Stickler et al., 1998; Papenfort and Bassler, 2016; Defoirdt, 2018; Roy et al., 2018; Williams et al., 2018). Anti-quorum sensing agents (anti-QSA) can interfere with or inhibit QS system by different ways, such as blocking signal molecule synthesis, degradation or modification, antagonizing or activating receptor, interfering with receptor-DNA binding or transcription activation, etc. (Mukherjee et al., 2018). When combating clinical pathogens, anti-QSA are different from traditional antibiotics in that they do not directly kill or inhibit bacteria but alter their behavior. Therefore, they can avoid bacterial resistance, reduce damage to normal flora and host tissue, enhance host immune system (LaSarre and Federle, 2013).

In recent years, QS have received extensive attention and research as a drug discovery target. There are many literature reviews and summaries on anti-QSA, but bibliometric analysis is still of great significance. Anti-QSA field involves multiple disciplines such as medicine, chemistry, materials science, microbiology, artificial intelligence (AI), etc. In this study, more than 1,700 articles were retrieved. Reading and analyzing such a large number of publications to understand the research history of anti-QSA discovery and extract research hotspots is a time-consuming and laborious task. Moreover, limited by one’s own experience, memory and adequacy of available literature, researchers may be forced to make subjective judgments about the historical picture of the development of the scientific field in traditional literature reviews. Bibliometric methods are based on scientific publication data to perform quantitative analysis and evaluation, which can use mathematical and statistical tools to process a large amount of information, such as publication counts, citation counts, impact factors, *h*-indexes, etc. Bibliometric methods can reveal the knowledge structure, research topics, research trends, research frontiers, etc. of a scientific field, without being limited by personal experience, memory and availability of literature, thus reducing the interference of subjective factors. Bibliometric methods can present the development history picture of a scientific field through data visualization and statistical interpretation, without relying on academic viewpoints or assumptions, thus providing a more objective and comprehensive description (Chen, 2004; Chen et al., 2014; Hou et al., 2018). In order to perform bibliometric analysis on the anti-QSA field, we collected publications on anti-QSA from the Web of Science Core Collection database, and used tools such as CiteSpace (Chen, 2006) and Alluvial Generator (Chen et al., 2014) to process and visualize the retrieved literature data. Our research objectives are: (1) to summarize the historical features of anti-QSA research; (2) to identify active topics in the research field; (4) to reveal emerging trends and hotspots for future research. We hope this paper can offer comprehensive and in-depth references for researchers in the anti-QSA discovery field and provide guidance and inspiration for further development of this field.

## 2 Methods

### 2.1 Study design

This paper used the bibliometric approach to study the current status, development, and future directions of discovering anti-quorum sensing agents against clinically relevant pathogens (Chen et al., 2012). The methods included publications analysis, co-citation visualization analysis, burst detection, timeline analysis, key concepts alluvial flow visualization, and structural variation analysis. The findings were reported following the Preferred Reporting Items for Systematic reviews and Meta-Analyses extension for Scoping Reviews guidelines (Supplementary Table S1) (Tricco et al., 2018).

### 2.2 Data collection and statistics

**Figure 1:**
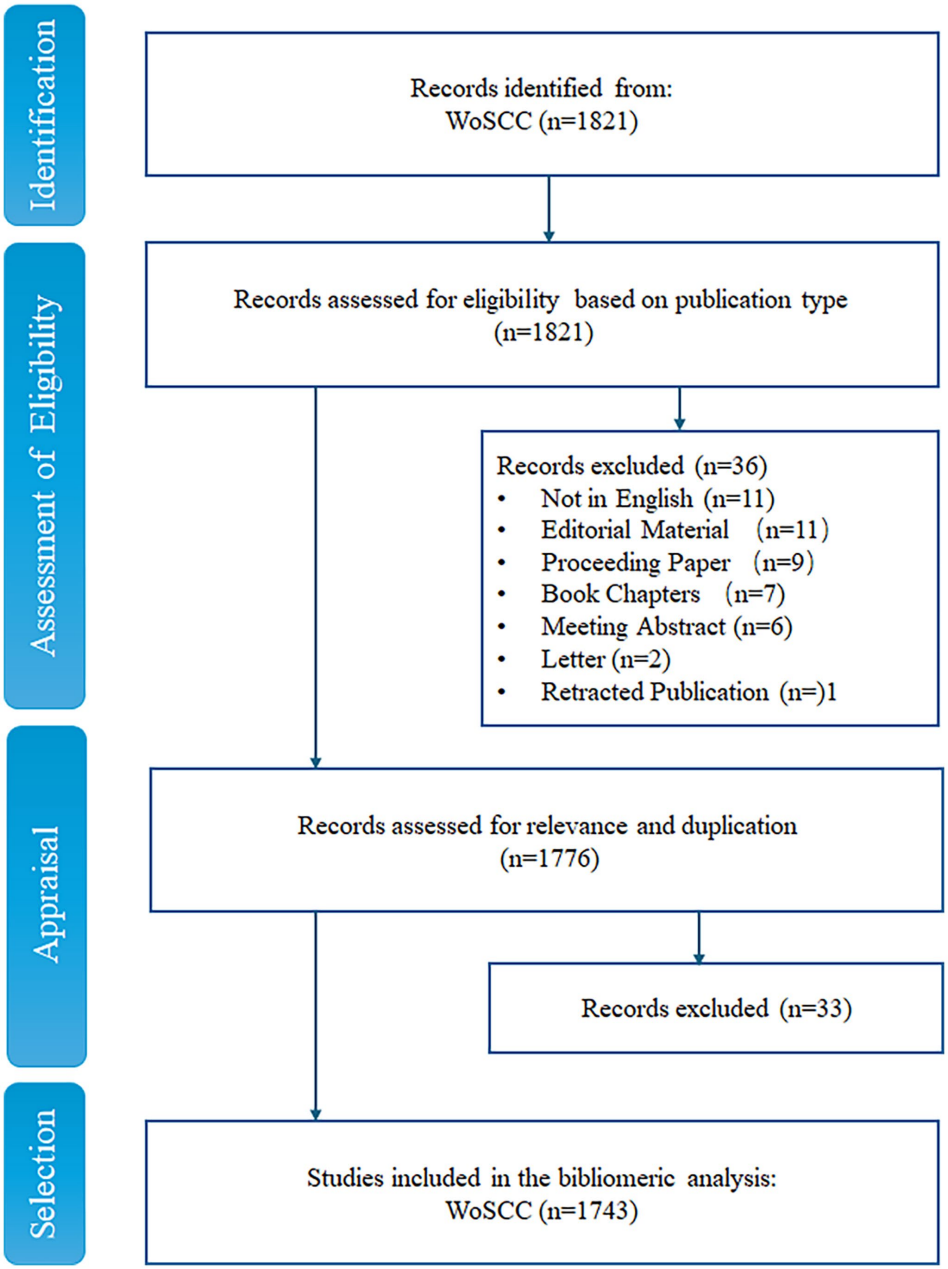
PRISMA framework.

We collected and analyzed the literature on anti-QSA from the Web of Science Core Collection (WoSCC), which is a comprehensive and authoritative database that covers over 12,000 influential academic journals in various disciplines (Tan et al., 2023). The search strategies were presented as follows: TS = (“anti-quorum sensing agents” OR ((“drug development” OR “drug discovery” OR “new drug*” OR “novel drug*” “preclinical trial*” OR clinical OR therapeutic) and (“quorum sensing” OR “Quorum Quenching”))), and Retracted Publication or Letter or Meeting Abstract or Book Chapters or Proceeding Paper or Editorial Material (Exclude – Document Types) and English (Languages). The search was performed on Wed May 03 2023 10:22:25 GMT + 0800 (China Standard Time) and yielded 1,821 publications. We applied the PICOS strategy to further screen the articles manually (Table 1) (Tricco et al., 2018). In this process, all papers retrieved from WoSCC were divided into relevant, uncertain, and excluded categories. Papers marked as unsure were screened by three of the authors (PP, LY, YH) and discussed to determine whether they should be included. According to the inclusion and exclusion criteria based on PRISMA guidelines (Tricco et al., 2018), 1,743 publications were finally obtained (Figure 1).

**Table 1:** Eligibility criteria using PICOS population, interventions, comparators, outcomes, and study design.

PICOS	Description
Participant	Anti-QSA against clinically relevant pathogens
Intervention	Evaluation approaches and methodologies for developing anti-QSA
Comparators	Antibiotic that directly kills or inhibits bacteria
Outcomes	Anti-QSA for treating infections avoiding bacterial resistance reducing damage to normal flora and host tissue enhancing host immune system
Study design	Systematic reviews including quantitative and qualitative studies

### 2.3 Data export and analysis

The content of data extraction was downloaded as plain text versions, which mainly includes literature title, cited references, published author, corresponding author, journal, country, institution, publication time, language, type, keyword, abstract, issue number, and page number of the article.

The aim of our study was to conduct a bibliometric analysis of the literature on anti-QSA and identify the development trends and research hotspots in this field. For this purpose, we applied Microsoft Office Excel 2019, and CiteSpace (v.6.4.R3 Advanced) to analyze 1,743 documents related to anti-QSA. CiteSpace was developed by Professor Chen Chaomei of Drexel University in the United States (Chen, 2006; Chen et al., 2012; Jie and Chaomei, 2016). It is a document visualization analysis software designed for bibliometrics analyses and data visualization. We employed CiteSpace to visually display the basic knowledge and hotspots of anti-QSA and predict its research frontiers. We also integrated an alluvial generator^1^ (Tan et al., 2023) with CiteSpace to generate an alluvial map to demonstrate the evolution and change of key conception in the anti-QSA field. Findings were reported in Preferred Reporting Items for Systematic reviews and Meta-Analyses extension for Scoping Reviews (PRISMA-ScR) guidelines.

For this study, the specific parameters of CiteSpace were set as follows: (1) Time Slicing: From January 1998 to April 2023; Years Per Slice: 1; (2) Term Source: Title, Abstract, Author, Keywords, and Keyword Plus; (3) Node Types: Category, Keyword, Term, and Reference (Burst analysis). Reference (Co-cited visualization). Term, and Key words (alluvial flow visualization), (4) Selection Criteria: G-index, *k* = 25.

Alluvial Generator was used to draw an alluvial flow map showing the change process of co-cited documents in the past 26 years 1998–2023. The modules of Terms and Key words cited in these years are colored, indicating that the key conception has received a high degree of attention in this time frame.

## 3 Results

### 3.1 The annual distribution of publications on anti-QSA

**Figure 2:**
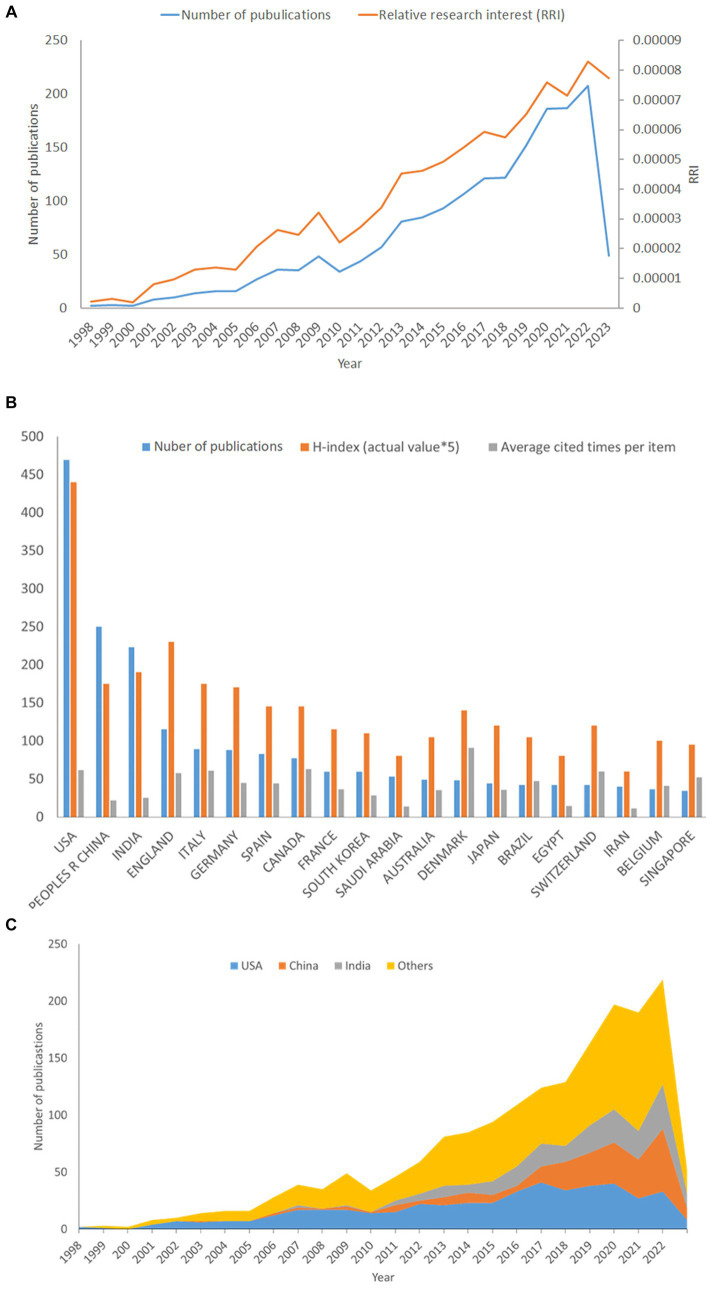
Contributions of different countries/regions to the publications on anti-QSA. The number of publications, *H*-index (×5), and Sum of citations (×0.05) in the top 20 countries or regions **(A)**; the number of publications worldwide and the time course of relative research interest (RRI) of anti-QSA **(B)**; the number of publications from the top three and other countries per year **(C)**.

From 1998 to 2023, we identified 1,743 publications that met our inclusion criteria. Figure 2A describes the number of publications in the anti-QSA field from 1998 to 2023, as well as Relative Research Interest (RRI), which was defined as the ratio of the number of publications in a specific research field to the number of publications across all fields per year in the WOSCC database (Xing et al., 2018). The number of publications per year peaked in 2022, with 208 publications. The RRI of anti-QSA fluctuated around 0.0022% before 2010, increased to 0.0083% in 2022. These trends indicate that the research interest in this field has been growing steadily.

### 3.2 Contributions of countries/regions to global publications on anti-QSA

The analyze results of WoSCC show that 93 countries/regions have carried out anti-QSA research. As shown in Figure 2B, the United States was the most productive country/region in anti-QSA research, with 469 publications (26.9%), followed by China with 250 publications (14.3%) and India with 223 publications (12.8%). Based on the Journal Citation Report from the WoSCC database, we found that all articles related to anti-QSA had been cited 75,511 times since 1998 (69,191 times without self-citations), with an average citation frequency of 43.28 times per paper. The United States received the most citations among all countries, making up 34.0% of the total citations, i.e., 28,669 times (27,842 times without self-citations), and achieving an H-index of 88. The number of citations from United Kingdom was 6,577 (6,449 times without self-citations) with an H-index of 46, and thus ranked second among all involved countries/regions. It has been widely accepted that the H-index reflects the scientific research impacts of a scholar or a country and indicates that a scholar or country has published H papers, each of which has been cited in other publications at least H times.

Figure 2C shows the annual number of publications from the top three countries and others. The United States was the first country to conduct research in this field, with 2 publications in 1998 and reaching its peak of 41 publications in 2017, after which the number of publications stabilized. China’s number of publications increased from 1 in 2003 to 55 in 2022. India’s number of publications increased from 2 in 2007 to 39 in 2022. The proportion of publications of China and India in this field increased rapidly in the past five years. The analysis above reveals that the research interest in this field has been continuously increasing. The United States, China, and India are the most productive countries in this field, while the United States and United Kingdom are the leading countries in terms of influence. China and India are the most rapidly growing countries in this field.

### 3.3 Journals with research publications on anti-QSA

**Figure 3:**
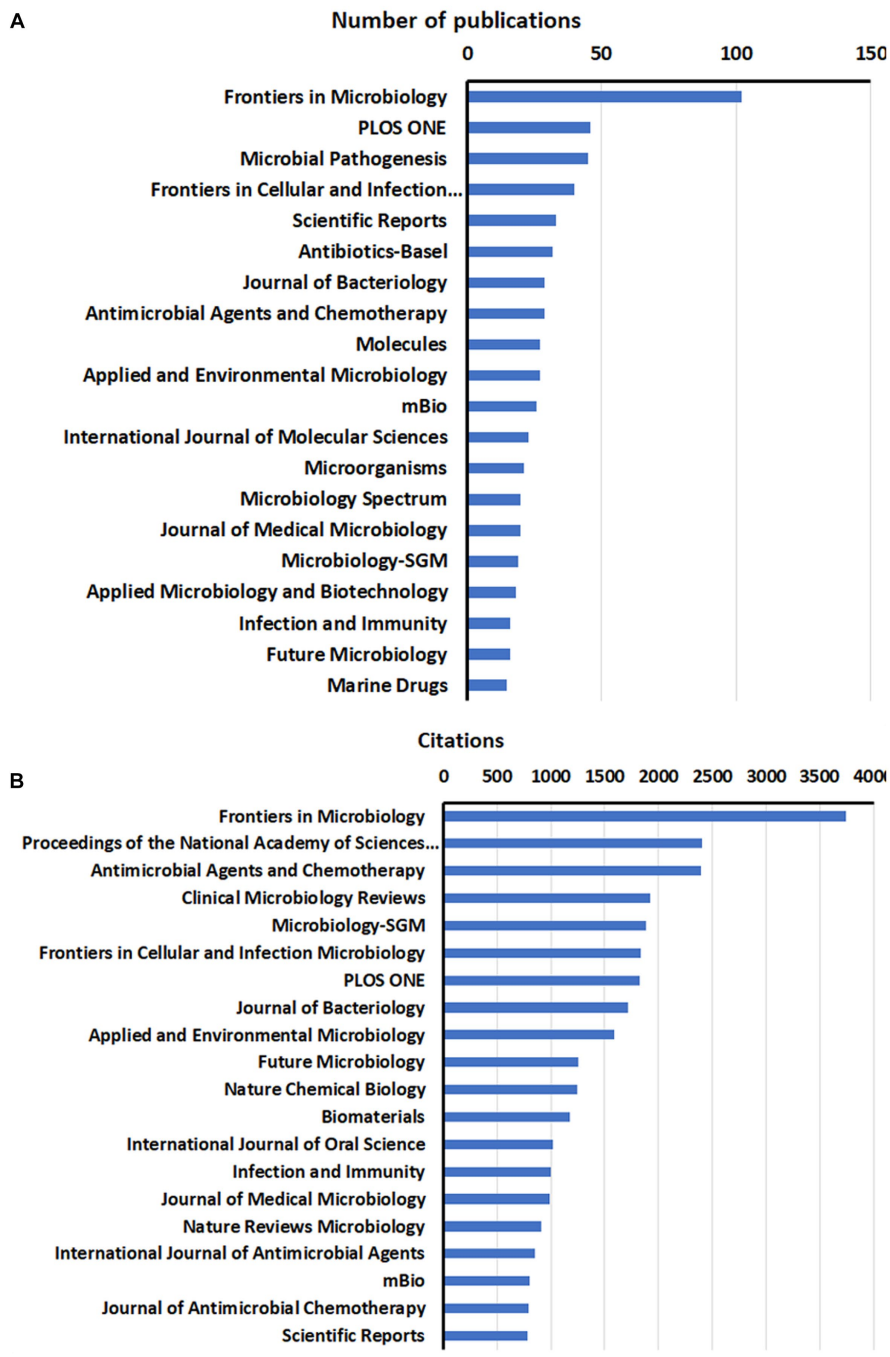
Distribution of the top 20 journals of anti-QSA research papers by publications **(A)**, and citations **(B)**.

The publications on anti-QSA were distributed among 558 journals, with the top 20 productive journals accounting for about one-third of the total papers (604, 34.65%). Figure 3 shows the ranking of these journals based on the number of documents and citations. Frontiers in Microbiology was the most prolific journal with 102 papers, followed by PLOS ONE with 46 papers, and Microbial Pathogenesis with 45 papers. The most cited journal was also Frontiers in Microbiology with 3,745 citations, followed by Proceedings of the National Academy of Sciences of the United States of America with 2,408 citations, and Antimicrobial Agents and Chemotherapy with 2,396 citations. These results indicate that, Frontiers in Microbiology is a leading journal in anti-QSA research.

### 3.4 Authors with publications on anti-QSA

In anti-QSA research, out of the total output, 152 papers were contributed by the top ten authors, accounting for 8.7% (Table 2). The most prolific authors were Williams and Camara, from the Nottingham University, who published 25 and 18 papers, respectively. Givskov M from Nanyang Technological University ranked third with 18 papers. This indicates that they might be the most active authors. The authors with the highest citations were Hoiby N, Givskov M, and Horswill AR, who received 2,028, 1,840 and 1,113 citations, respectively. This indicates that their research work received the most attention. They might be the most influential authors in the field.

**Table 2:** Top 10 authors with most publications on anti-QSA.

Rank	Author	Country	Affiliation	Publications (n)	Citations (n)
1	Williams P	United Kingdom	University of Nottingham,	25	1,036
2	Camara M	United Kingdom	University of Nottingham	18	583
3	Givskov M	Singapore	Nanyang Technological University	18	1840
4	Otto M	United States	National Institute of Allergy and Infectious Diseases	17	733
5	Shunmugiah KP	India	Alagappa University	17	611
6	Garcia-Contreras R	Mexico	Universidad Nacional Autonoma de Mexico	13	435
7	Horswill AR	United States	University of Colorado Anschutz Medical Campus	13	1,113
8	Bassler BL	United States	Princeton University	11	706
9	Hoiby N	Denmark	University of Copenhagen	10	2028
10	Ravi AV	India	Alagappa University	10	287

### 3.5 Institutions with research publications on anti-QSA

We analyzed the distribution of institutes to identify the top 20 institutions with the most publications in the research scope of anti-QSA. Table 3 shows the institute name, country, number of publications, and number of citations of these institutions. University of Nottingham was the most productive institution in anti-QSA research, with 37 publications, followed by University of Copenhagen with 32 publications. University of Washington ranked third in terms of productivity with 25 publications. They are the leading institutions in anti-QSA research strength and contribution. Harvard University, University of Copenhagen, and University of Nottingham had the highest number of citations, with 2,956, 2,930, and 2,722 citations respectively, indicating that their research was widely recognized and most influential in this field.

**Table 3:** Top 20 institutions with most publications in research scope of anti-QSA.

Rank	Institute	Country	Publications (n)	Citations (n)
1	University of Nottingham	United Kingdom	37	2,722
2	University of Copenhagen	Denmark	32	2,930
3	University of Washington (UW)	United States	25	1,188
4	Alagappa University	India	23	651
5	University of Maryland, College Park	United States	23	1765
6	Technical University of Denmark (DTU)	Denmark	22	2,234
7	Ghent University	Belgium	22	1,038
8	King Saud University	Saudi Arabia	19	325
9	National University of Singapore	Singapore	19	993
10	Nanyang Technological University	Singapore	18	1,006
11	Universidad Nacional Autónoma de México (UNAM)	Mexico	18	759
12	University of Iowa	United States	17	1,527
13	Aligarh Muslim University	India	16	1,031
14	National Institute of Allergy and Infectious Diseases (NIAID)	United States	15	896
15	Princeton University	United States	15	1,296
16	Panjab University	India	14	369
17	University of Pennsylvania (UPenn)	United States	14	707
18	Emory University	United States	13	681
19	Harvard University	United States	13	2,956
20	Massachusetts General Hospital	United States	13	1,551

### 3.6 Co-citation analysis of references on anti-QSA

**Figure 4:**
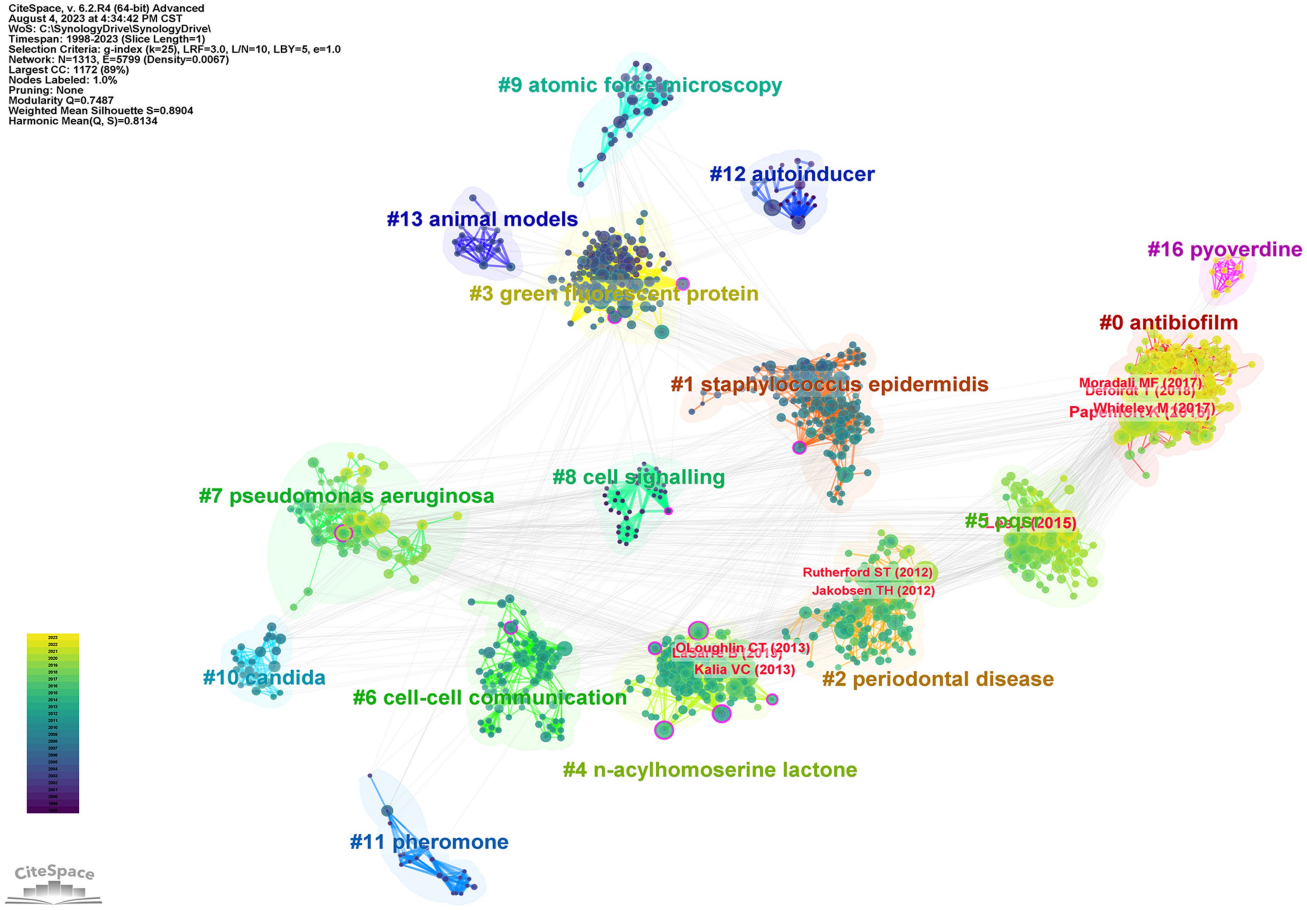
Co-citation network of anti-QSA literature from 1998 to 2023. The color gradient from deep blue (bottom) to yellow (top) indicates the year of publication. The figure shows the papers of 10 nodes with high intermediate centrality (purple rings), and the papers of the top 10 nodes by co-citation counts (red labels).

In this study, in order to characterize the emerging trends and patterns of anti-QSA research by the co-citation situation of references, we used CiteSpace, a visual analysis tool to construct co-citation networks (Garfield, 2004). The co-citation network graph (Figure 4) displays the co-citations among 1,313 references related to the anti-QSA research from 1998 to 2023, with 3,538 links. These references are represented as nodes in the network. The links between these nodes indicate the co-citation frequency between them, which is normalized by the total number of citations of each reference. The co-citation network was divided into a number of clusters of co-cited references using a modularity-based clustering algorithm, such that references are tightly connected within the same clusters, but loosely connected between different clusters. Each cluster is assigned an automatically generated cluster label, for example, #6 cell–cell communication and #9 atomic force microscopy. Clusters are numbered from #0 onwards.

Table 4 lists 17 major clusters by their size, namely, the number of members in each cluster. These clusters represent the coherence of themes within each cluster, although the degrees of coherence may vary widely across different clusters (Chen et al., 2012). The quality of a cluster is also reflected in terms of its silhouette score, which is an indicator of how close each member is to other members within the same cluster and how far away it is from members of other clusters. Silhouette values of compact clusters tend to close to 1. All of the clusters in Table 4 are highly compact, as shown by their silhouette scores. Each cluster is labeled by noun phrases from titles of citing articles of the cluster. Labels chosen by the log-likelihood ratio test method (LLR), which is a statistical method to measure the association strength between words and clusters, are used in the subsequent discussions.

**Table 4:** Major clusters of co-cited references.

Cluster ID	Size	Silhouette	Mean (Year)	Label (LSI)	Label (LLR)	Label (MI)
#0	230	0.882	2018	*Pseudomonas aeruginosa*	Antibiofilm	4-hydroxy-2-alkylquinolines
#1	138	0.839	2004	Biofilm	*Staphylococcus epidermidis*	Biomaterial-related infection
#2	126	0.824	2011	Antibiotic resistance	Periodontal disease	Biofilm exopolysaccharides
#3	120	0.899	2002	*Pseudomonas aeruginosa*	Green fluorescent protein	dsred fluorescent protein
#4	114	0.895	2010	*Pseudomonas aeruginosa*	*n*-acylhomoserine lactone	rat
#5	109	0.878	2015	*Pseudomonas aeruginosa*	pqsr	Quorum sensing receptor
#6	79	0.896	2007	*pseudomonas aeruginosa*	Cell–cell communication	Drug efflux transporters
#7	78	0.886	2014	Quorum quenching	*pseudomonas aeruginosa*	4-hydroxy-2-alkylquinolines
#8	39	0.994	1996	Quorum sensing	Cell signaling	Quorum sensing
#9	32	0.973	1999	*Staphylococcus aureus*	Atomic force microscopy	Quorum sensing
#10	24	0.992	2005	Cryptococcus	Candida	Quorum sensing
#11	22	0.962	1999	Quorum sensing	Pheromone	Quorum sensing
#12	21	1	1997	*Burkholderia cepacia*	Autoinducer	Quorum sensing
#13	17	0.981	2001	…	Animal models	Quorum sensing
#16	10	0.993	2019	*pseudomonas aeruginosa*	Pyoverdine	Quorum sensing
#17	5	1	2002	Gene regulation e.rex	tet	Quorum sensing

Some of the nodes in Figure 4 are surrounded by purple circles, which indicate their high betweenness centrality. Betweenness centrality is a measure of how often a node lies on the shortest path between any two other nodes in the network. This means that these nodes play a crucial bridging role between different stages of the development of anti-QSA field, as they connect different clusters of co-cited references that represent different research topics or subfields. These nodes also indicate the evolution process of the research field, as they reflect the changes and transitions of research themes and directions over time. Table 5 shows ten structurally essential references of the purple circle nodes in Figure 4. These works reported in the articles can be seen as landscape works in the context of our broadly defined area of anti-QSA (Chen et al., 2012).

**Table 5:** The highest betweenness centrality papers on anti-QSA.

Rank	Title	Author	Journal	Cluster	Publication year	Centrality value
1	Targeting QseC signaling and virulence for antibiotic development (Rasko et al., 2008)	Rasko DA	Science	#4	2008	0.23
2	Can resistance against quorum-sensing interference be selected? (Garcia-Contreras et al., 2016)	Garcia-Contreras R	ISME J	#6	2015	0.18
3	The *Pseudomonas aeruginosa* 4-quinolone signal molecules HHQ and PQS play multifunctional roles in quorum sensing and iron entrapment (Diggle et al., 2007)	Diggle SP	CHEM BIOL	#1	2007	0.18
4	Analysis of *Pseudomonas aeruginosa* 4-hydroxy-2-alkylquinolines (HAQs) reveals a role for 4-hydroxy-2-heptylquinoline in cell-to-cell communication (Déziel et al., 2004)	Deziel E	P NATL ACAD SCI USA	#7	2004	0.18
5	Discovery of antagonists of PqsR, a key player in 2-alkyl-4-quinolone-dependent quorum sensing in *Pseudomonas aeruginosa* (Lu et al., 2012)	Lu CB	CHEM BIOL	#4	2012	0.16
6	Identification of boronic acids as antagonists of bacterial quorum sensing in *Vibrio harveyi* (Ni et al., 2008)	Ni NT	BIOCHEM BIOPH RES CO	#4	2008	0.14
7	Quorum sensing in Acinetobacter: an emerging pathogen (Bhargava et al., 2010)	Bhargav NS	CRIT REV MICROBIOL	#4	2010	0.14
8	Quorum sensing in bacterial virulence (Antunes et al., 2010)	Antunes LCM	MICROBIOL-SGM	#8	2010	0.11
9	Quenching quorum-sensing-dependent bacterial infection by an N-acyl homoserine lactonase (Dong et al., 2001)	Dong YH	NATURE	#3	2001	0.11
10	Human paraoxonases (PON1, PON2, and PON3) are lactonases with overlapping and distinct substrate specificities (Draganov et al., 2005)	Draganov D	J LIPID RES	#4	2005	0.1

The size of a node in the co-citation network is proportional to the number of times it is co-cited. In Figure 4, the authors and publication years of the top 10 nodes with the largest size were labeled in red text, and Table 6 lists the information of these articles. The most cited articles are usually considered as landmarks due to their ground breaking contributions (Chen, 2006; Chen, 2012; Chen et al., 2012). Lee and Zhang (2015), Papenfort and Bassler (2016), and Whiteley et al. (2017) are the three papers with the highest co-citation.

**Table 6:** The top 10 most co-cited papers on anti-QSA.

Rank	Title	Author	Journal	Cluster	Publication year	Citations
1	The hierarchy quorum sensing network in *Pseudomonas aeruginosa* (Lee and Zhang, 2015)	Lee J	PROTEIN CELL	#5	2015	87
2	Quorum sensing signal–response systems in Gram-negative bacteria (Papenfort and Bassler, 2016)	Papenfort K	NAT REV MICROBIOL	#0	2016	87
3	Progress in and promise of bacterial quorum sensing research (Whiteley et al., 2017)	Whiteley M	NATURE	#0	2017	52
4	Exploiting quorum sensing to confuse bacterial pathogens (LaSarre and Federle, 2013)	LaSarre B	MICROBIOL MOL BIOL R	#4	2013	49
5	Quorum-sensing systems as targets for antivirulence therapy (Defoirdt, 2018)	Defoirdt T	TRENDS MICROBIOL	#0	2018	49
6	Quorum sensing inhibitors: an overview (Kalia, 2013)	Kalia VC	BIOTECHNOL ADV	#4	2013	49
7	A quorum-sensing inhibitor blocks *Pseudomonas aeruginosa* virulence and biofilm formation (O’Loughlin et al., 2013)	Oloughlin CT	P NATL ACAD SCI USA	#4	2013	42
8	*Pseudomonas aeruginosa* lifestyle: a paradigm for adaptation, survival, and persistence (Moradali et al., 2017)	Moradali MF	FRONT CELL INFECT MI	#0	2017	41
9	Ajoene, a sulfur-rich molecule from garlic, inhibits genes controlled by quorum sensing (Jakobsen et al., 2012)	Jakobsen TH	ANTIMICROB AGENTS CH	#2	2012	35
10	Quorum sensing inhibitors as anti-biofilm agents (Brackman and Coenye, 2015)	Rutherford ST	CSH PERSPECT MED	#2	2012	35

### 3.7 Burst detection

**Figure 5:**
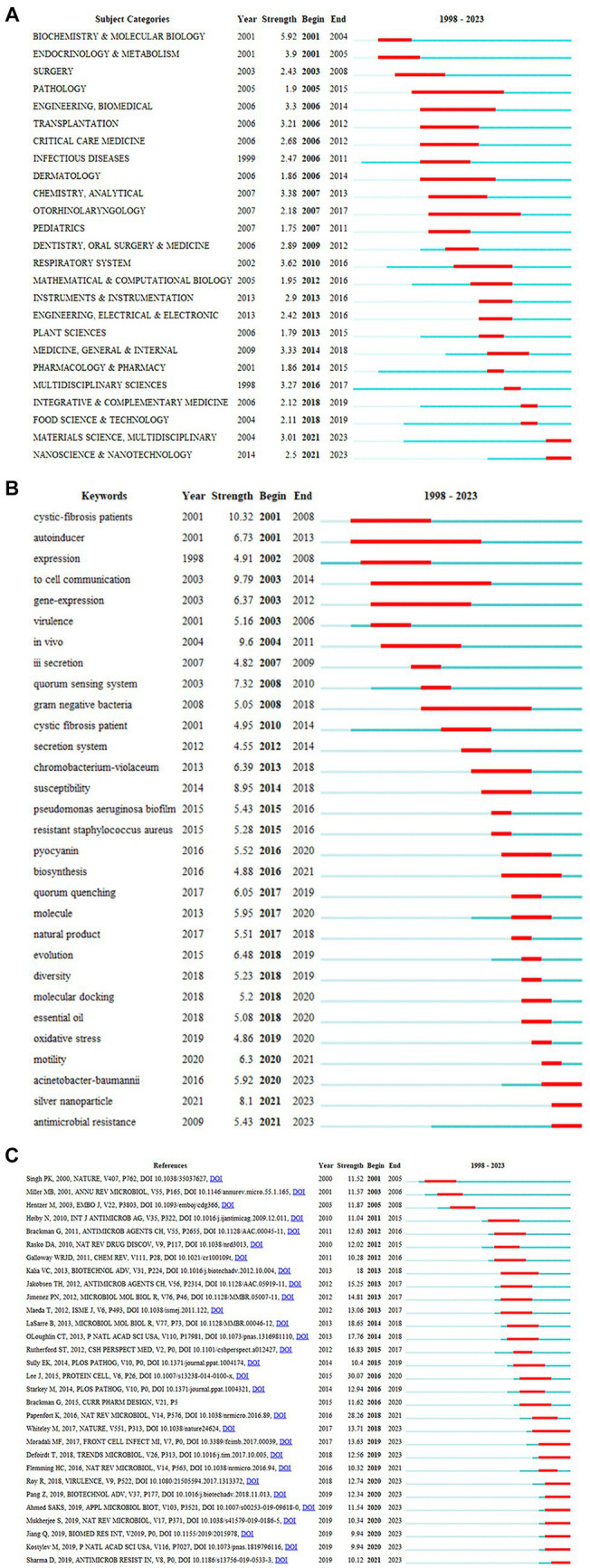
Burst detection **(A)** top 25 subject categories with the strongest citation bursts on anti-QSA research; **(B)** top 30 Keywords with the Strongest Citation Bursts; **(C)** top 30 references with the Strongest Citation Bursts on anti-QSA research. Year: Year of the first occurrence, Strength: Burst’s strength, Begin: Burst’s beginning year, End: Burst’s ending year.

Citation burst means that the number of citations increases in a short period of time, so burst detection can reveal the most active areas and emerging trends in the network (Xiao et al., 2022). The calculation method of burst strength in CiteSpace is based on Kleinberg’s burst detection algorithm (Chen, 2006). From 1998 to 2023, 26 out of 88 related subject categories experienced citation bursts. Figure 5A depicts the top 25 subject categories with high burst strength at different times. The blue line represents this time interval, the starting point of the dark blue line segment represents the year when a discipline began to appear, and the time span in which a subject category was found to have bursts is depicted as a red line segment with the beginning and end years of the bursts. For example, at the top of the list, the subject category BIOCHEMISTRY & MOLECULAR BIOLOGY had a burst period between 2001 and 2004, with a burst strength of 5.92. The hotspots before 2006 were only in biological and medical disciplines, such as BIOCHEMISTRY & MOLECULAR BIOLOGY (2001–2004), PATHOLOGY (2005–2015), and INFECTIOUS DISEASES (2006–2014). Since 2007, more and more disciplines outside of biology and medicine have received attention and started to burst, such as INSTRUMENTS & INSTRUMENTATION (2013–2016), PLANT SCIENCES (2013–2015), MULTIDISCIPLINARY SCIENCES (2016–2017), FOOD SCIENCE & TECHNOLOGY (2018–2019), MATERIALS SCIENCE MULTIDISCIPLINARY (2012–2023), and NANOSCIENCE & NANOTECHNOLOGY (2021–2023). From the temporal sequence of subject bursts, it can be seen that anti-QSA research has an increasingly obvious trend of multidisciplinary intersection. Multidisciplinary involvement in anti-QSA research has effectively advanced the directions of bioavailability, drug action mechanism, new structure drug molecule discovery, and so on.

A total of 55 keywords burst out at different time points. The top 30 keywords are shown in Figure 5B. The keyword with the highest burst strength was cystic-fibrosis patients, which burst between 2001 and 2008 with a strength of 10.32. This was followed by to cell communication, which burst between 2003 and 2014 with a strength of 9.79. *In vivo* burst between 2004 and 2011 with a strength of 9.6. Special attention was paid to the following keywords: silver nanoparticle, antimicrobial resistance, *Acinetobacter baumannii*. These keywords had a burst period until 2023. In the next few years, they might continue to burst.

A total of 273 publications had citation bursts, and Figure 5C displays the top 30 references (Tan et al., 2023). Lee and Zhang (2015) was the reference with the highest citation burst strength of 30.07 from 2016 to 2020, and it studied the QS network in *P. aeruginosa* that causes severe and persistent infections (Lee and Zhang, 2015). Papenfort and Bassler (2016) was the second strongest burst reference, with a burst strength of 28.26 from 2018 to 2021 (Papenfort and Bassler, 2016). In this reference, the authors examined the molecular mechanisms of QS in Gram-positive bacteria. LaSarre and Federle (2013) was the third strongest burst document, with the highest burst strength of 18.65, from 2014 to 2018 (LaSarre and Federle, 2013). This review discussed how to trick and stop bacterial pathogens by interfering with their communication, and suggested new ways to treat bacterial infections by blocking or altering their communication. In Figure 7, the citation bursts of ten references continue until 2023, and (Moradali et al., 2017; Whiteley et al., 2017; Pang et al., 2019) are the three references with the strongest bursts among the ten. Whiteley et al. (2017) reviewed the recent advances in QS research in the context of the extraordinary progress made in our understanding of the genetics, genomics, biochemistry, and signal diversity of bacterial communication. It also discusses the prospects of QS in drug discovery and social biology. Moradali et al. (2017) reviewed the diversity of mechanisms by which *P. aeruginosa* promotes its survival and persistence in various environments and particularly at different stages of pathogenesis. Pang et al. (2019) discusses antibiotic resistance *P. aeruginosa*. These papers are worth special attention, which may still be frequently cited in the next few years.

### 3.8 Timeline analysis of references on anti-QSA

**Figure 6:**
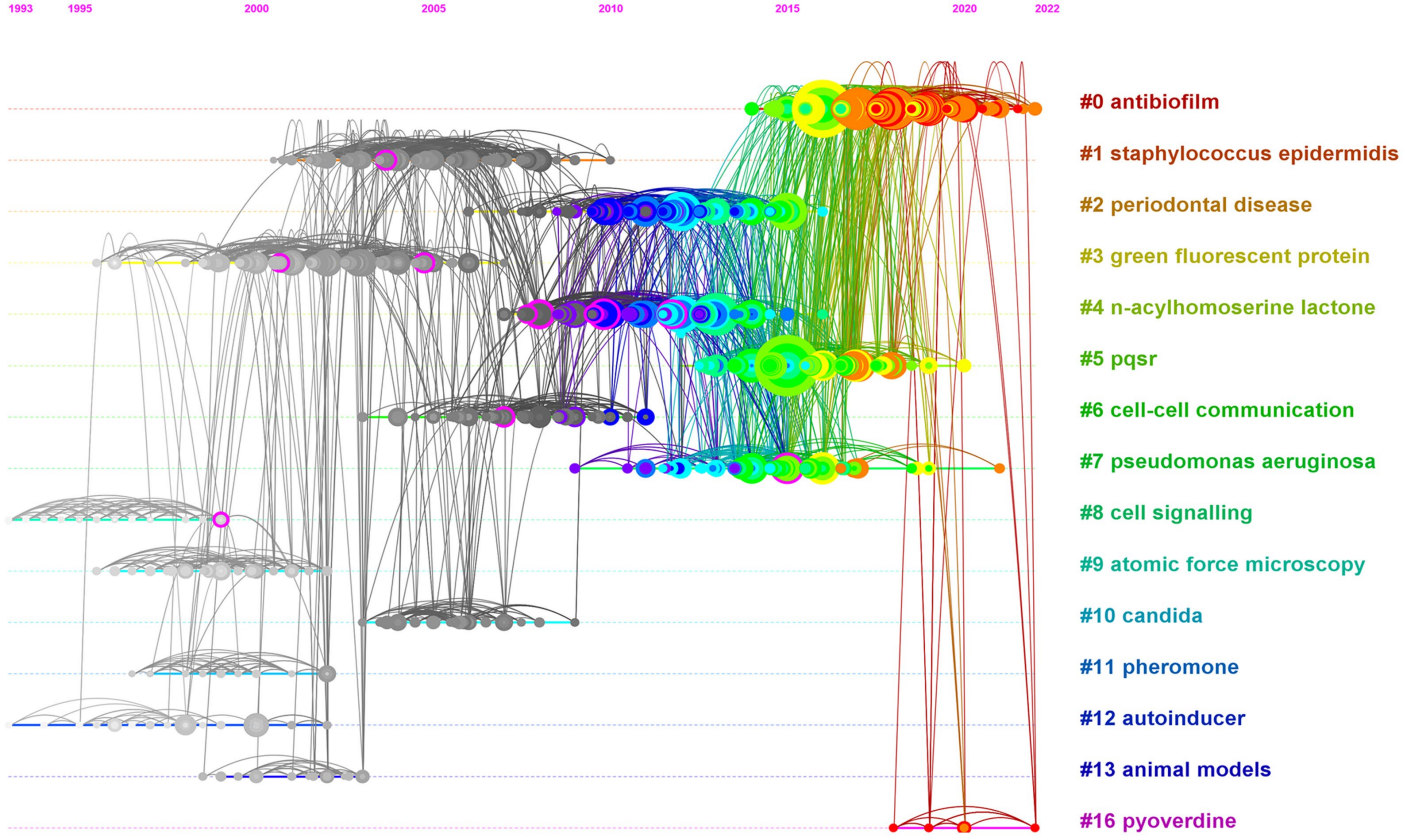
The citation timeline visualization.

A timeline visualization based on the citation span of the references is depicted to identify the emerging, classic, and outdated topics in the anti-QSA field (Figure 6) (Tan et al., 2023). The 17 clusters identified by co-cited analysis (Table 4) in each period were arranged from top to bottom according to their size. Each node in the figure represents a cited paper, and the nodes in the same cluster are arranged in chronological order according to the publication time of the corresponding papers on the same timeline. The area of the nodes is proportional to the number of co-citations, and the colors of different rings from inside to outside represent the citation span from early to recent. For example, orange and red rings indicate that the reference was cited in 2022 and 2023, respectively, which are very active references. A link between two nodes indicates that they were co-cited by the same article. The thicker the link, the more co-citations they have.

Figure 6 shows three types of clusters. The first type of clusters are mainly composed of gray nodes, such as Clusters #1 *staphylococcus epidermidis*, #2 periodontal disease, #3 green fluorescent protein, #4 n-acylhomoserine lactone, #5 pqsr, #6 cell–cell communication, #8 cell signaling, #9 atomic force microscopy, and #10 candida, #11 pheromone, and #13 animal models. According to the time scale (1993–2022) at the top of the graph, we can see that the average publication time of the articles in these gray clusters is before 2007. These clusters’ related research laid the foundation for the development of anti-QS research. However, these clusters have not produced new references in recent years, have not been cited by the latest published articles, and have few links with the clusters that contain the latest published articles. Therefore, these clusters are not active research topics anymore. There are two clusters, #2 antibiotic resistance, and 4# n-acylhomoserine lactone, which are mainly composed of blue and green nodes, with average publication time (2010–2011). These clusters are closely linked to other latest clusters that contain red tree ring nodes. However, due to their own lack of recent citations, these clusters are not the most active research topics. Simultaneously, in view of the close link between these two clusters and the clusters with gray nodes, cluster #4 and 5 can be considered as classic topics that bridge the inactive and active ones. The clusters that have the nodes with orange and/or red rings represent the most recently citation. These clusters have an average publication time of after 2015. This indicates that these clusters are active and are considered to be the new development trends in the research field. Cluster #0 antibiofilm has a citation span from 2014 to present, and contains a large number of nodes with large areas and orange-red rings (Luo et al., 2017; Moradali et al., 2017; Whiteley et al., 2017; Defoirdt, 2018; Jamal et al., 2018; Mukherjee et al., 2018; Roy et al., 2018; Ahmed et al., 2019; Pang et al., 2019). Since 2016, such nodes have appeared every year, which indicates that this is the most active and influential topic, and is very likely to continue this impact for a period of time in the future. #16 pyoverdine is a very new cluster, with a citation span from 2017 to 2023 (Gomila et al., 2018; Khan et al., 2019; De Oliveira et al., 2020; Mishra et al., 2022). Although it has few nodes and small areas, it may continue to grow and expand, and may become a future research hotspot. #7 *pseudomonas aeruginosa* (2009–2021) (Garcia-Contreras et al., 2016; Ali et al., 2017; Bzdrenga et al., 2017), and #5 pqsr (2012–2020) (Lee and Zhang, 2015; Dickey et al., 2017; Kitao et al., 2018; Mukherjee et al., 2018; Soukarieh et al., 2020), contain nodes with large areas and orange rings, indicating that they have a very large impact and are still active.

### 3.9 Key concepts alluvial flow visualization

**Figure 7:**
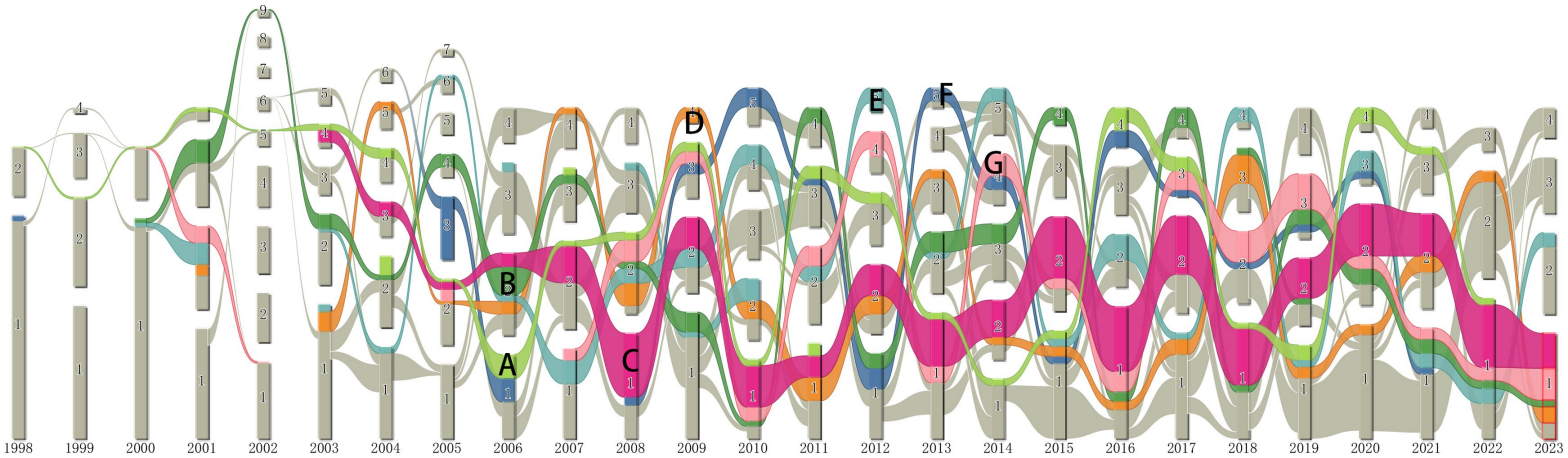
Terms alluvial map 1998–2023. **(A)** *E. coli*, **(B)** virulence, **(C)** *P. aeruginosa*, **(D)** virulence factor, **(E)** bacterial biofilm, **(F)** gene expression, **(G)** quorum sensing.

Alluvial flow maps can reveal temporal patterns in evolving networks (Rosvall and Bergstrom, 2010). CiteSpace was used to generate a series of individual networks of co-occurring terms, and they were fed into an alluvial generator, which generated Figure 7. It shows an alluvial flow visualization of terms extracted from the titles and abstracts of publications on anti-QSA (Soukarieh et al., 2018). These terms include keywords and noun phrases. The seven terms with the longest non-interrupted presence in the literature of anti-QSA are highlighted in Figure 7. These terms and their corresponding time spans and labels are: *Escherichia coli*, 1998–2022 (A), virulence, 2000–2023 (B), *P. aeruginosa*, 2003–2023 (C), virulence factor, 2003–2023 (D), bacterial biofilm, 2002–2023 (E), gene expression, 2002–2021 (F), quorum sensing, 2007–2022 (G). Although the flow (A) of *Escherichia coli* in Figure 7 does not directly extend to 2023, we attribute the flow (A) to the key concepts that have been persistent until the present day, considering that only the first four months of the current year were available for drawing this graph. Therefore, except for the flow of gene expression (F), which was interrupted in 2021, we can assume that all other key concepts in Figure 7 have continued to flow until now. These terms that have lasted the longest in the literature are not only key concepts in the research field, but also likely to be frequently used in future studies (Chen et al., 2014).

### 3.10 Structural variation analysis

Structural variation analysis (SVA) can identify articles that have a significant potential to transform the network of references, as they cite and are cited by novel and diverse references, while bridging different research topics (Burt, 2004; Chen, 2012). We applied the SVA function of CiteSpace to analyze the co-citation network of the literature and obtained the ten articles with the highest structural variation potential scores based on structural properties during the period from 1998 to 2023 (Table 7).

**Table 7:** Articles with the highest structural variation potential score (Δ Cluster linkage) based on structural properties during the period between 1998 and 2023.

Rank	Global cites	Δ Cluster linkage	Title	Citing article
1	87	135.21	The art of antibacterial warfare: Deception through interference with quorum sensing-mediated communication (Rampioni et al., 2014)	Rampioni G, 2014, BIOORG CHEM, V55, P60, DOI 10.1016/j.bioorg.2014.04.005
2	96	64.2	Targeting Antibiotic Tolerance, Pathogen by Pathogen (Meylan et al., 2018)	Meylan S, 2018, CELL, V172, P1228, DOI 10.1016/j.cell.2018.01.037
3	14	64.2	*In silico* identification of albendazole as a quorum sensing inhibitor and it’s *in vitro* verification using CviR and LasB receptors based assay systems (Singh and Bhatia, 2018)	Singh S, 2018, BIOIMPACTS, V8, P201, DOI 10.15171/bi.2018.23
4	90	50	Quorum sensing: a primer for food microbiologists (Smith et al., 2004)	Smith JL, 2004, J FOOD PROTECT, V67, P1053, DOI 10.4315/0362-028X-67.5.1053
5	50	17.86	Regulatory effects of macrolides on bacterial virulence: Potential role as quorum-sensing (Tateda et al., 2004)	Tateda K, 2004, CURR PHARM DESIGN, V10, P3055, DOI 10.2174/1381612043383377
6	211	11.8	Targeting mechanisms of *Pseudomonas aeruginosa* pathogenesis (Kipnis et al., 2006)	Kipnis E, 2006, MED MALADIES INFECT, V36, P78, DOI 10.1016/j.medmal.2005.10.007
7	41	0	*Pseudomonas aeruginosa* quorum-sensing signal molecule N-(3-oxododecanoyl)-L-homoserine lactone inhibits expression of P2Y receptors in cystic fibrosis tracheal gland cells (Saleh et al., 1999)	Saleh A, 1999, INFECT IMMUN, V67, P5076, DOI 10.1128/IAI.67.10.5076-5082.1999
8	183	0	Quorum sensing and the population-dependent control of virulence (Williams et al., 2000)	Williams P, 2000, PHILOS T R SOC B, V355, P667, DOI 10.1098/rstb.2000.0607
9	232	0	Lethal paralysis of *Caenorhabditis elegans* by *Pseudomonas aeruginosa* (Darby et al., 1999)	Darby C, 1999, P NATL ACAD SCI USA, V96, P15202, DOI 10.1073/pnas.96.26.15202
10	656	−20.66	*Pseudomonas aeruginosa* Lifestyle: A Paradigm for Adaptation, Survival, and Persistence (Moradali et al., 2017)	Moradali MF, 2017, FRONT CELL INFECT MI, V7, P, DOI 10.3389/fcimb.2017.00039

**Figure 8:**
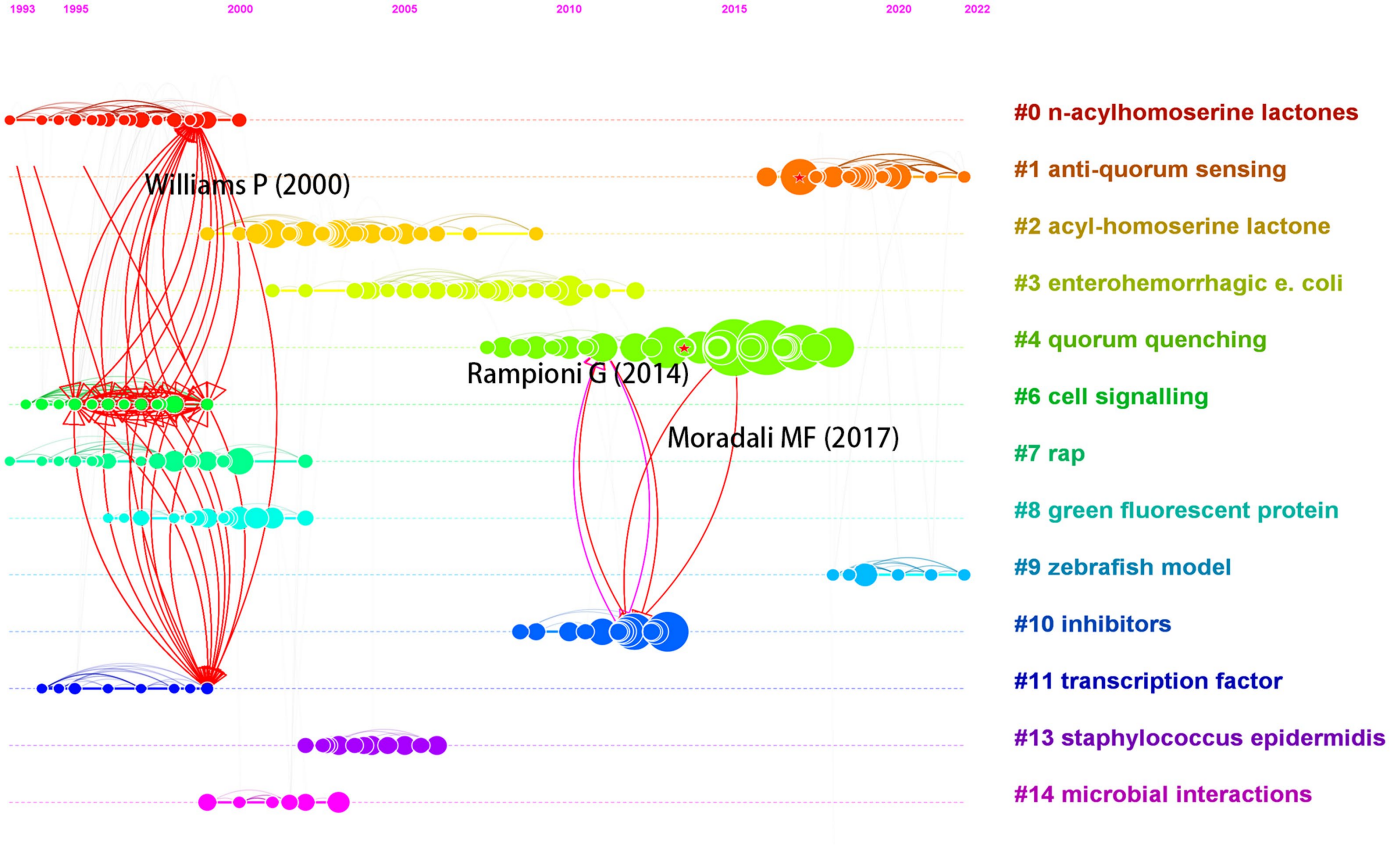
Visualization of bridging multiple clusters by three articles with high structural variation potential.

Figure 8 shows the different clusters in the bridging co-citation network of three representative articles. Williams et al. (2000) bridged three clusters: #0 n-acylhomoserine lactones, #6 cell signaling, and #11 transcription factor. This article, which was cited 183 times, discussed QS and population-dependent control of virulence. It bridged the most clusters among the three articles. Rampioni et al. (2014) and Meylan et al. (2018) had the highest structural variation potential score and bridged clusters #4 quorum quenching and #10 inhibitors. This article, which was cited 87 times, discussed QS systems as multiple targets for drugs that attenuate virulence. Moradali et al. (2017) also bridged clusters #4 and #10. With 656 citations, it was the most cited article among the top 10 articles with the highest structural variation potential. These articles with high transformative potentials may receive more citations in the future.

## 4 Discussion

In WoSCC, 8,268 authors from 1,040 institutions in 96 countries published 1,743 articles that met the inclusion criteria, and their articles were published in 558 scientific journals. The analysis of annual publications, RRI index, citations, and H-index showed that the anti-QSA research field is still in a rapid development stage. Anti-QSA has great potential to combat resistant pathogens, which may be an important reason for the increasing attractiveness of this field. The United States, China, and India were the countries/regions with the most publications; the United States and the United Kingdom were the countries/regions with the highest influence in this field. China and India were the fastest-growing countries/regions in this field (Figure 2). Frontiers in Microbiology, PLOS ONE, and Microbial Pathogenesis were the top three journals in terms of publication quantity. Frontiers in Microbiology, Proceedings of the National Academy of Sciences of America, and Antimicrobial Agents and Chemotherapy had the highest citations and were influential (Figure 3). The most prolific authors were Williams and Givskov, and the authors with the highest citations were Hoiby et al. (Table 2). The three most productive research institutions were the University of Nottingham, the University of Copenhagen, and the University of Washington (UW). The top three institutions in terms of influence were Harvard University, the University of Copenhagen, and the University of Nottingham (Table 3). Scholars can pay attention to the research results of countries/regions, institutions, and authors with high productivity and influence. This is one of the effective ways to analyze and mine the current research hotspots and frontiers, and to explore an efficient research path to publish more high-quality research results in recognized and influential authoritative journals in the future.

Using the co-citation network, we can understand the development of Anti-QSA research. The literature is divided into 17 major clusters of different years, sizes and themes according to their co-citation situation (Figure 4). The 10 articles with the highest Centrality value in the whole network are regarded as landscape articles. These articles were published before 2015 and are distributed in 6 clusters. #4 contains the most landscape papers, with 5 papers (Draganov et al., 2005; Ni et al., 2008; Rasko et al., 2008; Bhargava et al., 2010; Lu et al., 2012), and clusters #1 (Rasko et al., 2008), 3 (Diggle et al., 2007), 6 (Garcia-Contreras et al., 2016), 7(Déziel et al., 2004), and 8 (Antunes et al., 2010) each have one paper. The average publication time of cluster #4 is 2010, and it is automatically named as *n*-acylhomoserine lactone. The papers with high Centrality value in the cluster are all about finding or designing compounds that can interfere with or inhibit the binding of signal molecules and receptors. The landscape attribute of the cluster 4# is very obvious, it links a conceptual cluster in one time period with one in another time period and can be seen as a bridge extending from earlier to more recent ideas. Time line analysis further confirms that it is the boundary between active and inactive clusters.

The co-citation network also helps to identify the most important research directions and interests in the field. The co-citation frequency reflects the importance and influence of the literature. The top ten co-cited papers (Table 6) are distributed in cluster #0 with 4 papers (Papenfort and Bassler, 2016; Moradali et al., 2017; Whiteley et al., 2017; Defoirdt, 2018). This cluster is named by the algorithm as biofilm, and the common research theme of the papers in the cluster is anti-biofilm and anti-virulence drug development, with strong comprehensiveness. The specific research contents include: analysis of the complex QS network involving multiple signal molecules in *P. aeruginosa*; structure, biosynthesis, secretion, transport, recognition and degradation of signal molecules; screening, identification, validation and mechanism of action of QS targets; design, synthesis, evaluation and mechanism of action of anti-biofilm/virulence compounds. The concepts involved include *P.aeruginosa*, QS signals, QS regulation, QS targets, anti-virulence strategies, etc. (Papenfort and Bassler, 2016; Jamal et al., 2018; Remy et al., 2018; Roy et al., 2018; Pang et al., 2019; Saipriya et al., 2020). There are three top ten cited papers in cluster #4 (*n*-acylhomoserine lactone) (Kalia, 2013; LaSarre and Federle, 2013; O’Loughlin et al., 2013). The theme of this cluster is drugs targeting signal molecules and their receptors. The common research is to inhibit the pathogenicity and resistance of bacteria by interfering with signal molecules. Frequently occurring terms include *P. aeruginosa*, drug molecule design and synthesis, LasR and RhlR system, pathogenicity and resistance (Dickey et al., 2017; Kitao et al., 2018; Soukarieh et al., 2018). #4 is also the cluster with the most papers with top ten centrality value, but none of the highly cited papers in the cluster have the highest centrality value, which may be a phenomenon worth thinking deeply about. There are two highly cited papers in cluster #2 (periodontal disease) (Jakobsen et al., 2012; Brackman and Coenye, 2015). The research theme of this cluster is to inhibit biofilms related to dental plaque as an entry point, to carry out activity evaluation and screening of anti-biofilm natural products from food and microorganisms, and to find anti-QSA. Frequently occurring terms include biofilm, natural product, food, *P. aeruginosa*, *Staphylococcus. aureus*, resistance. There is one top ten cited paper in cluster #5 (pqsr). The common research theme in this cluster is the receptor of signal molecules. The main research contents include: structure, function, regulation and inhibition mechanism of receptors of different types of QS signal molecules, as well as their relationship with bacterial virulence and biofilm. *P. aeruginosa*, signal pathway, and anti-infection are frequently occurring concepts in this cluster (Soukarieh et al., 2020; Schutz et al., 2021; Soukarieh et al., 2021; Boopathi et al., 2022; Hamed et al., 2023).

The time line visualization can help us further understand the changes of research interests over time and identify the most active research directions at present. Figure 6 shows active periods of 17 clusters with most co-citations identified by co-cited analysis. Cluster #0 antibiofilm is a very new cluster, with papers published from 2014 to 2022, and an average publication year of 2018. This cluster is not only the cluster with the most nodes, but also includes a large number of nodes with large area and red or orange edge (highly cited papers that are still frequently cited until 2023), and there are new emerging papers almost every year within its time span, which is undoubtedly the most active research direction of anti-QSA at present. As introduced in the previous co-cited network section, the research theme of Cluster #0 is about anti-biofilm/anti-virulence drug. Cluster #5 pqsr (2012–2020), whose research theme is the receptor of signal molecules, has an average year of 2015 and some nodes with large area and orange edge, have the characteristics of an active research direction. #7 is automatically labeled as *pseudomonas aeruginosa* by the algorithm, with an average publication year of 2014. The nodes with large area and red and orange edge in the cluster indicate that this cluster is also related to the active research topic. The research in this field focuses on two important human pathogens, *S. aureus* and/or *P. aeruginosa*, which both have complex QS systems, regulating biofilm formation, toxin secretion, antibiotic resistance, etc. (Garcia-Contreras, 2016; Chang et al., 2017). #16 is automatically assigned pyoverdine as the title. The average publication time of the cluster is 2019, and all nodes are red. The theme of the cluster is the biosynthesis, mechanism of action, and interference or inhibition of pyoverdine as a representative virulence factor by compounds from different sources or structures. The concepts frequently involved in the cluster include *P. aeruginosa*, siderophore virulence factors, biofilm formation, marine natural products, antimicrobial peptides, plant extracts, metal nanoparticles and chitosan, etc. #16 has a very recent emergence time and has the characteristics of an emerging research field. Although it currently includes few nodes with small area, it has a great potential to develop into a new hotspot in the future. The above four clusters are most active topics that may remain active for a period of time in the future. Clusters #2 periodontal disease, #4 *n*-acylhomoserine lactone, etc., contain a large number of nodes with yellow-green edges. Although they lack recent citations, they have a large number of co-citations with the latest clusters, and are relatively active clusters. Clusters #1, 3, 6, 8–13, etc., are all composed of gray-white nodes, no longer have newer nodes appearing, and have very few links with the most active cluster. They are topics that have laid a foundation for anti-QSA development, but they are no longer the active research directions.

The burst of subject categories, keywords, and cited references (Figure 5) is a valuable indicator of the most active research topics at various levels of granularity. The analysis of subject bursts shows that in the past 26 years, the main disciplines of the research frontier have changed from BIOCHEMISTRY & MOLECULAR BIOLOGY, ENDOCRINOLOGY & METABOLISM ENGINEERING, BIOMEDICAL, INFECTIOUS DISEASES, to CHEMISTRY, ANALYTICAL, RESPIRATORY SYSTEM, MULTIDISCIPLINARY SCIENCES. The trend of interdisciplinary research is very obvious. In the analysis of keyword bursts, pathogenic bacteria, biofilm, resistance, quorum sensing inhibitory active molecules are hot terms throughout the research history. After 2016, keywords of emerging fields such as marine natural products, and nano materials appeared bursts, which also reflected the continuous expansion of the scope of active fields. Nanomaterials have a large burst intensity and extend to 2023, which may be a hot spot for research in the future. Nanotechnology can solve the limitations of traditional anti-QSA methods, such as side effects, delivery difficulties, etc., and improve antibacterial efficacy and safety (Adu et al., 2022; Jeevitha et al., 2022; Kumar et al., 2022; Zahmatkesh et al., 2022; Zhang et al., 2022). Natural products, especially marine natural products are important resources for developing anti-QSA. Its advantage lies in the huge structural diversity, which provides a vast source of materials for finding and developing anti-QSA with diverse, unique, innovative and synergistic features (Borges and Simoes, 2019; Zhao et al., 2019; Chen et al., 2020; Nawaz et al., 2021; Hemmati and Kazerooni, 2022; Lahiri et al., 2023).

Structural variations measure the potential of a transformative research idea. Table 7 shows the 10 articles with the highest transformative potential based on the Δ Cluster linkage, which may influence the future research direction and breakthrough. The concepts involved in the articles include *P. aeruginosa*, resistance, quorum sensing, food, anti-virulence drugs and receptors. One of the papers in Table 7 mentions computer-aided drug design (CADD) methods to explore anti-QSA. Singh and Bhatia (2018) shows the use of molecular docking techniques to predict the binding mode and interaction of albendazole with bacterial QS receptor proteins (Singh and Bhatia, 2018). Molecular docking is a typical CADD method, which can use optimization algorithms in AI to find the optimal ligand-receptor binding conformation. In addition, the paper uses COMSTAT to analyze the morphological characteristics of biofilms, such as thickness, density, surface area, etc. COMSTAT is a software based on image processing and analysis, which can use image recognition technology in AI to extract biofilm information (Hemmati and Kazerooni, 2022; El-Naggar et al., 2023; Limayem et al., 2023). The application of AI technology is undoubtedly the trend of future development of anti-QSA research and development. AI can overcome the limitations and challenges of traditional drug discovery, such as the lack of drug targets, the complexity of drug molecules, the difficulty of drug synthesis, the evaluation of drug safety and efficacy, and individual differences of drugs. ChatGPT is a major breakthrough in AI. It is an AI language model based on natural language generation technology. It can communicate with humans, generate different types of texts, and even discover potential drug targets. It has the potential to change the mode of future anti-QSA discovery (Corsello and Santangelo, 2023; Ruksakulpiwat et al., 2023; Savage, 2023).

This study analyzed the literature on anti-QSA discovery from a bibliometric perspective, which is relatively systematic and objective. However, this study also has some limitations. First, the publications in this study only came from WoSCC database, without considering other databases such as CNKI. Second, the language type of the publications was limited to English, and important publications in other languages may have been overlooked. Third, all data in this study were mainly extracted by software, unlike systematic reviews that are subjected to rigorous screening by independent authors, so there may be some omissions in the retrieval results. If readers are particularly interested in some specific details in the field, it is recommended to conduct further related analysis.

## 5 Conclusion

Anti-QAS research is a rapidly growing field, and the United States is the largest contributor and the most influential country. The frontier disciplines shifted from biomedicine-related fields to food, materials, natural products, multidisciplinary and other fields. The research interests and directions mainly focused on anti-biofilm/anti-virulence drug development, signal molecules and their receptor-targeted drugs, anti-biofilm natural products, and regulation mechanisms of signal molecule receptors. The current most active research areas are anti-biofilm/anti-virulence drug development, interference and inhibition of virulence factors, interference of signal molecules and their receptors, and complex QS networks of major pathogenic microorganisms. Emerging fields such as nanomaterials, marine natural products, AI and others are worthy of special attention and may become hotspots in the future. This article is a relatively systematic and objective overview and analysis of the anti-QSA drug discovery field, which may help scholars quickly and intuitively understand the current status and trends of the field, locate key articles, and explore future research directions and breakthroughs.

## Data availability statement

The raw data supporting the conclusions of this article will be made available by the authors, without undue reservation.

## Author contributions

PB: Data curation, Formal analysis, Investigation, Writing – original draft. YL: Data curation, Formal analysis, Writing – original draft. YJ: Supervision, Writing – review & editing. DW: Supervision, Writing – review & editing. XZ: Supervision, Writing – review & editing. WF: Supervision, Writing – review & editing. YH: Conceptualization, Methodology, Project administration, Supervision, Writing – review & editing, Writing – original draft.

## Footnotes

^1^ http://www.mapequation.org/apps/AlluvialGenerator.html
