# Supplementary material for: A bibliometric analysis on discovering anti-quorum sensing agents against clinically relevant pathogens: current status, development, and future directions
Source: Front Microbiol. 2023 Nov 30;14:1297843. doi: 10.3389/fmicb.2023.1297843 (PMC10720721; doi:10.3389/fmicb.2023.1297843)
Supplement: Supplementary file 1 [file Table_1.docx]

Supplementary Material

**Table S1. Composition and nutrient levels of the basal diet.**

| Ingredients | Dosage (g) | Additives | Dosage (g) |
| --- | --- | --- | --- |
| Secondary corn | 436.3 | Methionine | 1.5 |
| Soybean meal | 284 | Lysine | 1.25 |
| Corn protein powder | 9.7 | Secondary Sub-Powder | 7.68 |
| Jiangzhe Red Wheat 3090 | 200 | 438 Multidimensional | 0.36 |
| First-grade fishmeal | 15.5 | Vitamin C | 0.1 |
| Grease | 6.5 | Poultry mine | 1.3 |
| Stonewash | 10.5 | Soda | 1.5 |
| Gypsum Powder | 5 | Lerisol | 0.5 |
| Calcium hydrogen phosphate (bone source) | 12.5 | Salt | 2.35 |
|  |  | Choline chloride | 1.3 |
|  |  | Olsenium | 0.05 |
|  |  | Enzyme Complex 8411 | 0.25 |
|  |  | Phytase | 0.15 |
|  |  | Lipase | 0.15 |
|  |  | Xylanase | 0.06 |
|  |  | Threonine | 0.5 |
|  |  | Insect and fly cleaner | 0.5 |
|  |  | Corunsun | 0.5 |
